# A review of polydactyly and its inheritance: Connecting the dots

**DOI:** 10.1097/MD.0000000000032060

**Published:** 2022-12-16

**Authors:** Dalal K Bubshait

**Affiliations:** a Department of Pediatrics, College of Medicine, Imam Abdulrahman Bin Faisal University, Dammam, Saudi Arabia.

**Keywords:** congenital deformities, inheritance, polydactyly

## Abstract

This study collects what is known about the inheritance underpinnings of syndromic and non-syndromic polydactylies and highlights dactyly presentations with unknown genetic roots. This review summarizes the current information and genetics-enhanced understanding of polydactyly.

**Background:**

There is a frequency of 0.37 to 1.2 per 1000 live births for polydactyly, which is also known as hyperdactyly. It is characterized by the presence of extra fingers. Polydactyly is caused by a failure in limb development, specifically the patterning of the developing limb bud. The phenotypic and genetic variability of polydactyly makes its etiology difficult to understand. Pre-axial polydactyly, central polydactyly (axial), and postaxial polydactyly are all examples of non-syndromic polydactyly (ulnar). An autosomal dominant disorder with varying penetrance that is mostly passed down via limb development patterning abnormalities.

**Method:**

A comprehensive search of MEDLINE/PubMed and other databases was followed by an evaluation of the relevant papers, with a particular focus on those published between 2000 and 2022.

**Results:**

Of 747 published article related to Polydactyly from MEDLINE/PubMed search, 43 were from the last 10 years and were the focus of this review.

**Conclusion:**

Polydactyly is one of the most frequent congenital hand malformations. PAP is more common than PPD, whereas central polydactyly is very uncommon.

## 1. Introduction

Congenital deformities of the hands, such as polydactyly, are extremely common. As a common limb-related birth defect,^[1]^ polydactyly, also known as hyperdactyly, has an incidence between 0.37 and 1.2 per 1000 live births, depending on race.^[2]^ Polydactyly is a limb abnormality defined by the presence of additional fingers on the hands.^[3]^ A deficiency in limb development, namely in the patterning of the developing limb bud, is the cause of polydactyly. Isolated cases or syndrome-related manifestations are also possible.^[4]^ It can occur as an isolated disease (non-syndromic polydactyly) and play a part in syndromic polydactyly also. Most common types of polydactyly are the pre-axial polydactyly (PPD) and the postaxial polydactyly (PAP) therefore rare types are: Meso-axial or central polydactyly; mirror image polydactyly; palmer; and dorsal polydactyly, etc.^[5,6]^

The present need is to classify newly found genes, which might help physicians and researchers comprehend molecular etiology, the pathways involved, and speedy genetic diagnostics that could manage medical obstacles. As polydactyly is linked to hundreds of different syndromic disorders, expanding our knowledge of newly identified pathways and their interlinked genes may aid in future therapeutic treatments. Furthermore, particular pathogenic variations in the causative gene may induce the phenotype in question, but diverse variants in modifier genes may explain phenotypic variability. This study highlights the current state of knowledge on polydactyly, including methods for polydactyly classification, and inheritance for syndromic and non-syndromic polydactyly.

Method: Using the search phrase “polydactyly of hand” in MEDLINE and PubMed yielded a total of 747 papers in the medical literature. There were a total of 80 documents found once the search was limited to the time period between January of 2000 and July of 2022. Articles from 43 publications were considered for inclusion in the review after irrelevant articles were removed.

## 2. Classification

Polydactyly is classified into 2 broad categories such as syndromic and non-syndromic polydactyly.

### 2.1. Non-syndromic polydactyly

Temtamy–McKusick classified 4 pre-axial, 2 postaxial, and complicated polydactylous entities, all of which segregated autosomally dominant.^[7]^ Goldstein et al suggested extending the Temtamy-McKusick method by including subtypes (10 pre-axial, 9 postaxial, 4 high-degrees, and 7 complicated kinds).^[8]^ Castilla et al^[2]^ proposed that hand PAP were distinct conditions.^[9]^ Thumb and hallux polydactylies were diverse in genetics according to Orioli and Castilla.^[10]^ The Temtamy–McKusick polydactyly category is the most often used among geneticists and dysmorphologists. In this view, the 3 primary polydactylies are pre, postaxial, and complicated, each of which has several subtypes.^[7]^ Types of mutations in genes associated with non-syndromic PAP are shown in Table 1.

**Table 1:** Types of mutations in genes associated with non-syndromic PAP (HGMD).

Phenotype	Gene name	cDNA	Type of mutation
Polydactyly	GLI3	c.2690C > G	Missense
		c.668G > A	
		c.1633C > A	
		c.1658G > A	
		c.1673C > T	
		c.1698C > G	
		c.2844G > A	
		c.3534G > C	
		c.366C > G	Nonsense
		c.559G > T	
		c.919C > T	
		c.2799C > A	
		c.3640C > T	
		c.4507C > T	
		c.1498-1G > C	Splice Site
		c.2104-3C > A	
		c.733delA	Small deletions
		c.1274_1275delGC	
		c.1653delA	
		c.1687_1694delTTGAAAAC	
		c.1798delA	
		c.2211delT	
		c.2867delG	
		c.4038delG	
		c.3496delA	
		c.1180_1181insT	Small insertions
		c.1286dupC	
		c.1513dupC	
		c.2054dupA	
		c.565_567delCCCinsTCT	Small indels
		c.819_820delTAinsC	
Polydactyly and syndactyly	GLI3	c.739C > T	Nonsense
Pre-axial polydactyly	GLI3	c.2252delA	Small insertions
		c.1320dupT	
		c.714T > A	Nonsense
Pre-axial polydactyly IV	GLI3	c.3383delA	Small deletions
		Exon 4	Gross deletions
Postaxial polydactyly	GLI3	c.1627G > A	Missense
		c.1180delCinsTT	Small indels
	IQCE	c.895_904del10	Small deletions
		c.1350_1353delAGAG	
	KIAA0825	c.50T > C	Missense
		c.2173A > T	Nonsense
		c.591dupA	Small insertions
Postaxial polydactyly A	FAM92A1	c.478C > T	Nonsense
	ZNF141	c.1421C > T	Missense
	DACH1	c.563G > A	Missense
	IQCE	c.395-1G > A	Splice site
	GLI3	c.3997C > T	Nonsense
		c.2292delA	Small deletions
		c.4141delA	
		c.3568dupG	
		c.3855dupC	
Postaxial polydactyly B	GLI3	c.2372delC	Small deletions
Postaxial polydactyly A/B	GLI3	c.3707delG	Small deletions
		c.2179G > A	Missense
		c.1927C > T	Nonsense
	Gli1	c.816G > T	Missense
		c.1133C > T	
		c.1139G > A	
		c.883C > T	
		c.934T > C	
		c.946G > A	
		c.1064C > A	
		c.985A > T	Nonsense
		c.847A > T	

cDNA = complementary DNA, DACH1 = Dachshund Homolog 1, IQCE = IQ Motif Containing E, ZNF141 = Zinc Finger Protein 141.

### 2.2. Postaxial polydactyly (PAP)

PAP (ulnar polydactyly), is the most prevalent and is characterized by the presence of extra fingers on the ulnar side of the hand. The spectrum of additional digits ranges from basic skin tags to fully formed, functioning digits. Many postaxial supernumerary digits are formed of soft-tissue components alone.

### 2.3. PAP classification according to Stelling and Turek’s categorization^[11]^

• Type 1: soft-tissue structures duplication,
• Type 2: osseous structures duplication,
• Type 3: completely duplicated ray (including the metacarpal) is involved.

### 2.4. PAP classification according to Temtamy and Mckusick^[7]^

The most common type of polydactyly is PAP which involves the 5th digits and, approximately 77% to 87% of the total polydactyly. The incidence of this type of polydactyly varies along ethnic lines and is particularly common in Africa. PAP can be divided into 6 genetic types. Polydactyly is related to disease phenotype, chromosome location, genetic mode, and related pathogenic genes.^[12]^ According to the extra digit(s) PAP can be further classified:

• Type A: well-formed digits with an osseous link to the rest of the hand, and
• Type B: incompletely formed, nonfunctional digits connected by a skin bridge alone.^[7]^

In the United States, type A PAP is less prevalent than type B and occurs at similar rates among African children of origin.^[13]^ The prevalence of PAP is 1 to 2/1000 live births, with some differences in ethnic groups.^[14–16]^ A fully-fledged extra digit characterizes PAPA on the ulnar or fibular side with either a dominant inheritance model or autosomal recessive inheritance pattern. The frequency of type B polydactyly is 1/100 to 300 live births in African-American children and 1/1500 to 3000 in white children.^[11,13]^ Seventy percent of PAP (autosomal dominant) in African children are bilateral and are not often linked with other disorders.^[13]^ PAP occurs more often unilaterally, is sporadically inherited, and may be coupled with other congenital hand anomalies in children who are not of African heritage. This type of polydactyly is characterized by the duplication of a finger from the ulnar side of the hand or a toe from the fibular side of the feet.^[17]^ In the absence of strong family history, the patient should be sent to a geneticist since PAP is related to around 15 distinct disorders, including type 1 orofacial-digital syndrome and Meckel syndrome. PAP is identified and evaluated in several places, including the newborn nursery and the offices of pediatricians, family doctors, dermatologists, and hand surgeons. If osseous structures are felt during the examination, radiographs of the afflicted hand should be acquired; however, radiographs are often not required for duplication of just soft-tissue portions.

Base on genetics, PAP-A is further classified as PAPA1-PAPA11 subtypes. There are 11 different types of PAP documented in the literature.^[16,18]^

### 2.5. PAP-A classification

Based on genetic background and symptoms, PAP has been divided into eleven different groups. IQ Motif Containing E, FAM92A, GLI3, KIAA0825, and Dachshund Homolog 1 gene mutations have been linked to non-syndromic PAP subtypes (PAPA1-PAPA11).^[18–20]^

**PAPA1**: GLI3 gene heterozygous variations on chromosome 7p14.1 are responsible for the common digit deformity known as PAPA1.^[21]^ In non-syndromic cases, an additional digit that is well-developed is inherited in an autosomal dominant pattern on the side of the fifth metacarpal. The gene for GLI3 encodes a protein that is a member of the C2H2-type zinc finger proteins subclass of the Gli family that functions as a transcription factor that binds to deoxyribonucleic acid and mediates the sonic Hedgehog (Shh) pathway.^[21]^

**PAPA2**: It is characterized by clinical traits or phenotypes that overlap with PAPA1 and the development of isolated PAP, either bilateral or unilateral. PAP that is isolated and dominant has been linked to PAPA2. PAPA2 is located in the 13q21 to q32 region of the chromosome and has an autosomal dominant inheritance pattern. Investigation of a 27-month-old kid with surgically excised bilateral PAP of the hands revealed heterozygosity for a de novo inverted duplication in the long arm of chromosome 13.^[22]^

**PAPA3**: For the first time, PAPA3 was discovered in a 6-generation Chinese family and demonstrated an autosomal dominant form of PAP-A and PAP-B.^[23]^ The afflicted individuals also showed additional bilateral postaxial digits that were well-developed and functioning but had varying expressions. The disease loci for PAPA3 were identified in chromosomal bands 19p13.2-p13.1 using particular markers, D19S1165 and D19S929. The responsible gene has not yet been found.

**PAPA4**: The characteristic features of PAPA4 include PAP and partial cutaneous syndactyly (in some individuals). PAPA4 was described in a 6-generation Dutch family with a total of 31 members, including 11 afflicted and 20 normal people. PAPA4 has an autosomal dominant inheritance pattern.^[24]^ The phenotypes of syndactyly and polydactyly showed varying degrees of expression. PAP and syndactyly in this kind of PAPA are genetically diverse with substantial penetrance, according to the mapping of its location on chromosome 7q22 (Galjaard et al, 2003).^[24]^ There is no known gene that causes the condition.

**PAPA5**: Clinical features of PAPA5 include bilateral PAP in both the upper and lower limbs, cutaneous syndactyly (in a small number of cases), hallux deformity, and forked fifth metatarsals in the foot.^[17]^ Additionally, radiographs of the feet showed partly duplicated fifth metatarsals as well as additional toes with distinct metatarsophalangeal and interphalangeal joints. Using the Rutgers combined-linkage physical map, PAPA5 was located on chromosome 13q13.3-q21 between microsatellite markers D13S1288 and D13S632, encompassing a 17.87 cM region.

**PAPA6**: It exhibits an autosomal recessive inheritance pattern. A large bilateral, well-formed duplicated fifth digit that is deviated to either the ulnar or the radial side, as well as a duplicated distal phalanx of the fifth finger in certain people, are characteristics of those who are afflicted. PAPA6 has been linked to a homozygous missense mutation (c.1421C > T; p.Thr474Ile; rs587776959) in the Zinc Finger Protein 141 gene on chromosome 4p16.3.^[25]^

**PAPA7**: is an autosomal recessive disease that was initially identified in a Pakistani family with PAPA that only affected the lower limbs.^[26]^ The IQ Motif Containing E gene included a homozygous splice site variant (c.395-1G>; p.Gly132Valfs*22), which was further confirmed using a mini-gene splice experiment.^[26]^

**PAPA8:** Three different families with biallelic variants in the GLI1 gene (MIM 165220) linked to PAPA8 were described by Palencia-Campos et al.^[27]^ Additional characteristics in the described individuals were PAP, atrial septal abnormalities, minor nail dysplasia, small stature, or genu valgus. Ellis–Van Creveld syndrome (EVC), a condition brought on by weakened Hh signaling, coexisted with these developmental abnormalities. The dosage impact of GLI1 mutations is linked to the diversity in phenotypic presentation.^[27]^ The GLI1 gene has undergone many different mutations that have been linked to PAPA8. The GLI1 gene, which codes for a protein with 1106 amino acids, is found on chromosome 12q13.3.

**PAPA9**: Non-syndromic PAP in both the upper and lower limbs is a feature of PAPA9. On chromosome 8q21.13-q24.12, the homozygous variations of the FAM92A gene (MIM 618219) are linked to this autosomal recessive disease.^[18]^ No other phenotypes, such as dental malformations, skin deformities, hearing anomalies, or any linked syndromic anomalies, were present in the afflicted individuals.^[28]^

**PAPA10**: Bi-allelic missense mutations in the K AA0825I gene, is found on chromosome 5q15.^[19]^ The protein that is encoded by KIAA0825 (also known as C5orf36) has a frameshift (c.591dupA; p.[Gln198Thrfs*21]) and a nonsense variant (c.2173A > T; p.[Lys725*]), and its function is uncertain.^[19]^ Novel homozygous biallelic missense variant (c.50T > C, p.[Leu17Ser]; g.81528T > C) in the exon 3 of the KIAA0825 (NM 001145678.2) was discovered in a consanguineous family from Pakistan with 2 affected members. The described patients showed features such bilateral proximal phalangeal arthritis of the fingers (PAPA), bilateral camptodactylous fifth toes, and large knuckle pads.^[29]^

**PAPA11**: A non-syndromic recessive condition, PAPA affects both the upper and lower limbs, and minor syndactyly affects the second and third toes. In a single Pakistani family, PAP has recently been linked to a biallelic missense mutation (c.563G > A; p.Cys188Tyr; NM 080760.5) in the Dachshund Homolog 1 gene, which is located on chromosome 13q21.33.^[20]^ The parents and the rest of the family were all normal and had no skeletal deformities.^[20]^

### 2.6. Polydactyly in the preaxial axis

**Figure 1.**
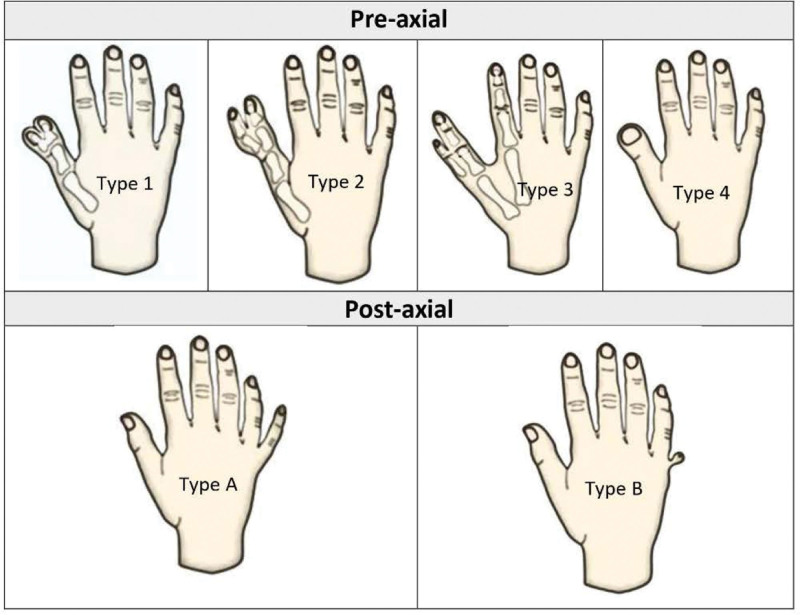
Phenotypic presentation of types of pre-axial polydactyly (PDD) and postaxial polydactyly (PAP).

Preaxial polydactyly (PPD) is less prevalent than PAP, with a frequency of 0.08% to 1.4% per 1000 live births.^[30]^ In PPD, a wide range of duplications may be seen, from a normal thumb to an entirely duplicated ray. To varying degrees, tendons, bones, ligaments, and blood vessels are abnormally formed in the hands of people who have had their thumbs duplicated. The phenotypic presentation of pre-axial polydactyly and PAP is shown in Figure 1.

Wassel classification is the most extensively utilized classification method for preaxial polydactyly.^[31]^ Working distally to proximally, the method allocates odd numbers to bifid phalanges and metacarpals (e.g., type I is a bifid distal phalanx) and even numbers to joint-level duplications (e.g., type IV involves duplicated proximal and distal phalanges and a shared metacarpophalangeal joint). The pattern is disrupted by any type VII triphalangeal thumb. Several additional changes, such as triphalangeal subtypes,^[32]^ type IV subtypes, and systems integrating symphalangism, deviation, and triplication, have been developed, but the original classification is still frequently used to guide therapy and facilitate communication between surgeons. Wassel type IV (i.e., duplicated proximal and distal phalanx) is the most prevalent variation of preaxial polydactyly,^[33]^ affecting 40% of individuals. Type II thumb is the second most prevalent form, affecting around 20% of people,^[33]^ Thumb polydactyly often occurs unilaterally and randomly, although it may also be related to Holt–Oram syndrome, Fanconi anemia, or Rubinstein-Taylor syndrome. 1 in 100,000 babies is affected with Holt-Oram syndrome, which is characterized by a range of heart problems and a variety of hand deformities, with thumb hypoplasia being the most frequent.^[33]^ Patients with Holt-Oram syndrome are often identified beforehand of surgeon assessment. Fanconi anemia is a rare disorder that affects one in 300,000 births and is characterized by failure of the bone marrow. One of 83 individuals with preaxial polydactyly in one study had Fanconi anemia.^[34]^ One in 125,000 babies is affected with Rubinstein–Taybi syndrome, which is marked by growth retardation, dysmorphic facial features, intellectual incapacity, and duplication of the distal phalanges of the thumb.^[35]^ The assessment of a syndromic patient includes a comprehensive history and physical examination, with special emphasis on the presence of additional congenital anomalies. PPD is further divided into many categories.

• Preaxial polydactyly type 1 (PPD1): Duplication of a biphalangeal thumb.^[15]^
• Preaxial polydactyly type 2 (PPD2): Thumb has an extra middle phalanx with an abnormally long and thin first metacarpal, having epiphyses at both ends.^[15]^
• Preaxial polydactyly type 3 (PPD3): The index finger is usually duplicated, one or two triphalangeal digits replace the thumb.
• Preaxial polydactyly type 4 (PPD4): The thumb is duplicated mildly, and the distal phalanxes show radial deviation or with a broad and bifid thumb.^[36,37]^

### 2.7. Central polydactyly

Central polydactyly is distinguished by the index duplication, long, and ring fingers, as well as the radial aspect of the little finger, inside the hand. It is far less prevalent than PAP or pre-axial polydactyly, ranging from 5% to 15% of all polydactylies,^[32,38]^ and is commonly linked with syndactyly or cleft hand.^[35]^ In the biggest cohort of individuals with central polydactyly, 55% had a family history of comparable hand variations, according to Wood.^[13]^ However, in the study of 12 Japanese individuals with central polydactyly, Tada et al^[38]^ could not identify a single patient with a family history could not be identified. Although the number of individuals with extraskeletal signs was minimal in both sets, the rarity of the disorder necessitates genetic consultation. Reconstruction is often difficult and must be adapted to the duplication’s peculiarities. Based on the features of the duplicated finger, central polydactyly is categorized into 3 subtypes.^[38]^

• Type I: Duplications do not have osseous or ligamentous attachments to the neighboring finger.
• Type II: Duplications contain osseous (normal) and soft-tissue structures inside the duplicated digit and share a joint, bifid metacarpal, or phalanx with the neighboring finger.
• Type IIa: No concurrent syndactyly.
• Type IIb: The presence of simultaneous syndactyly.
• Type III: Duplicate ray with a metacarpal that is fully grown.

The technique which is used in central polydactyly is describing plantar and dorsal advancement flaps has been described by Allen.^[39]^ When the second ray is duplicated, laterally based dorsal and plantar flaps are developed, beginning at the medial margin of the forefoot and extending distally along the great toe to the level of the first web space. When the fourth ray is duplicated, medially based dorsal and plantar flaps are developed, beginning at the lateral margin of the foot and extending distally along the small toe to the level of the first web space. Retrospective case series of 22 patients with 27 feet with central polydactyly, treated by the above-mentioned technique, reported excellent results.^[40]^

### 2.8. Polydactyly genetics

Polydactyly genes tend to influence particular physiological areas, such as the zone of polarizing activity, which regulates limb morphology and positional identity.^[41,42]^ It vanishes around the 44th day of embryonic development when the maturation of phalanges.^[43]^ Apart from the zone of polarizing activity (which produces fibroblast growth factor 8 (FGF8), which is located on the posterior embryo, the apical ectodermal ridge, which is located on the dorsal-ventral line and also produces FGF8. HOX genes, hedgehog pathways (sonic Hedgehog (SHH) and Indian Hedgehog (IHH), FGFs, bone morphogenetic proteins, and cartilage-derived morphogenetic proteins all play a significant role in limb development.^[44]^ The SHH signaling system is essential for limb development.^[45]^ 15 SHH is influenced by or influences various transcription factors, including HAND2, GLI3, ALX4, and some bone morphogenetic proteins antagonists (formin and gremlin), alterations which have resulted in polydactyly. 10 SHH cannot lead to proper limb development if it is interrupted.^[46]^

Chondrocyte differentiation and ossification are two processes that the IHH signaling pathway may influence.^[47]^ Gremlin and FGF8 expression are also restricted in the final stages of digit formation, which is why IHH is suppressed by fibroblast growth factor receptors (FGF)^[47]^ which function at a different time than WNTs and FGFs, which are involved in the final stages of mesenchymal ossification^[48]^; later, in the final stage of digit formation, the gremlin is down-regulated and FGF8 expression is restricted.^[49]^ GLI3 is implicated in several disorders, and a GLI3 missense mutation caused polydactyly.^[50]^ Preaxial polydactyly has been linked to GLI3 and SHH.

**Figure 2.**
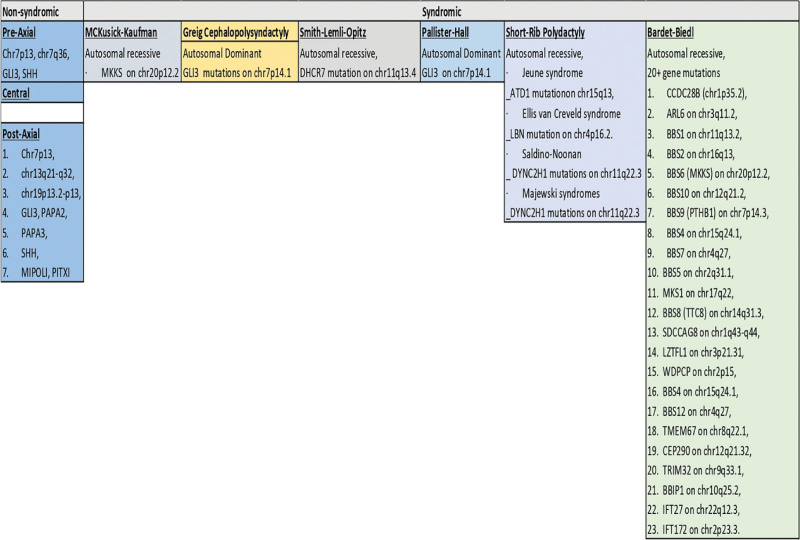
Genetics of syndromic and non-syndromic polydactylies.

Syndactyly and cleft hand are common complications of central polydactyly. It most often occurs as a syndrome. Finally, PAP is distinguished by a hypoplastic or completely grown little finger, is often bilateral, and is frequently accompanied by foot deformities.^[51]^ It is linked to GLI3 and PAPA2 and PAPA3. It has also been linked to SHH mutations, mirror-image Polydactyly gene, and PITXI (Fig. 2).^[51]^ The genes and signaling pathways that cause polydactyly are not single or completely independent, often interact with each other, or even have a regulatory effect. The relationship between genes, pathways, and disease is symmetrically involved. The introduction of the Turing machine had always played the important role in the original understanding of the mechanism of Hox gene family regulating polydactyly.^[7,52,53]^

### 2.9. Syndromic polydactylies

#### 2.9.1. Bardet–Biedl disease

Bardet–Biedl disease is characterized by syndactyly and polydactyly in the fingers and toes. It may be caused by mutations in at least 20 genes. These disorders are often inherited in an autosomal recessive or digenic recessive manner.^[54]^ In one of the 78 patients, only 9 had a follow-up period of >5 years and of the 118 feet, only 4 feet were complaints recorded in the long term due to abnormalities.^[55,56]^

#### 2.9.2. Syndrome of Greig cephalopolysyndactyly

It is also a limb abnormality affecting the limbs, head, and face. Polydactyly of the fingers or toes, as well as cutaneous syndactyly, are common. It is autosomal dominant and is caused by GLI3 mutations on chr7p14.150.^[54]^

### 2.10. Mckusick–Kaufman disease

Polydactyly, heart abnormalities, and genital abnormalities are all features of this condition. It is related to MKKS on chromosome 20p12.2^[57]^ and autosomal recessive disorder.

### 2.11. Pallister–Hall syndrome

A multitude of developmental anomalies, such as polydactyly and cutaneous syndactyly, differentiate it. Pallister–Hall syndrome is an autosomal recessive condition caused by the gene GLI3 on chromosome 7p14.1.^[58]^

### 2.12. Polydactyly with short ribs

A small thorax and preaxial polydactyly describe short-rib polydactyly syndromes. “Jeune syndrome (ATD1 on chromosome 15q13),” “Ellis van Creveld syndrome (LBN on chromosome 4p16.2),” “Saldino–Noonan and Majewski syndrome (DYNC2H1 mutations on chr11q22.3)” are among them. These syndromes are all defined by polydactyly. These disorders are often autosomal recessive and digenic.

### 2.13. Smith–Lemli–Opitz disease

There are several parts of the body that are affected by it. Syndactyly affects predominantly the 2nd and 3rd toes. It is autosomal recessive and caused by the DHCR7 gene on chromosome 11q13.4.^[59]^

### 2.14. Syndrome of triphalangeal thumb-polydactyly

PPD and PAP, isolated syndactyly, complicated polydactyly, and triphalangeal thumbs are known as triphalangeal thumb-polydactyly. Hands are usually the most impacted. This condition is linked to LMBR1 on chromosome 7q36.3 and is inherited autosomal. The resulting replicative limb, or polydactyly, is caused by early activation of abnormal SHH signals, which can be influenced by the external environment or caused by the body’s signal disorders.^[60]^

In recent years, with the further development of sequencing technology, some studies have found that de novo mutations of important genes related to polydactyly can also lead to polydactyly in the next generation.^[61]^

## 3. Conclusion

Polydactyly is one of the most frequent congenital hand malformations. PAP is more common than PPD, whereas central polydactyly is very uncommon. According to current research, genes, signaling pathways, and other biological components commonly interact and have regulatory effects on one another. As a result, the relationship between genes, pathways, and other biological variables should be thoroughly investigated in order to comprehend the underlying illness process. To disclose the pathophysiology of a disease, researchers should thus engage in inter-disciplinary thought, hypothesis, and even experimental design.

## Acknowledgments

The author is very thankful to all the associated personnel in any reference that contributed in/for the purpose of this research.

Author contributions

**Writing – original draft:** Dalal K Bubshait.

**Writing – review & editing:** Dalal K Bubshait.
